# Knockdown of *Hsc70-5/mortalin* Induces Loss of Synaptic Mitochondria in a *Drosophila* Parkinson’s Disease Model

**DOI:** 10.1371/journal.pone.0083714

**Published:** 2013-12-30

**Authors:** Jun-yi Zhu, Natalia Vereshchagina, Vrinda Sreekumar, Lena F. Burbulla, Ana C. Costa, Katharina J. Daub, Dirk Woitalla, L. Miguel Martins, Rejko Krüger, Tobias M. Rasse

**Affiliations:** 1 Junior Research Group Synaptic Plasticity, Hertie-Institute for Clinical Brain Research, University of Tübingen, Tübingen, Germany; 2 Graduate School of Cellular & Molecular Neuroscience, University of Tübingen, Tübingen, Germany; 3 German Center for Neurodegenerative Diseases, Tübingen, Germany; 4 Department of Neurodegenerative Diseases and Hertie-Institute for Clinical Brain Research, University of Tübingen, Tübingen, Germany; 5 Cell Death Regulation Laboratory, MRC Toxicology Unit, Leicester, United Kingdom; 6 Department of Neurology, St. Josef-Hospital, Ruhr-University Bochum, Bochum, Germany; 7 Werner Reichardt Centre for Integrative Neuroscience, University of Tübingen, Tübingen, Germany; Oregon Health & Science University, United States of America

## Abstract

Mortalin is an essential component of the molecular machinery that imports nuclear-encoded proteins into mitochondria, assists in their folding, and protects against damage upon accumulation of dysfunctional, unfolded proteins in aging mitochondria. Mortalin dysfunction associated with Parkinson’s disease (PD) increases the vulnerability of cultured cells to proteolytic stress and leads to changes in mitochondrial function and morphology. To date, *Drosophila melanogaster* has been successfully used to investigate pathogenesis following the loss of several other PD-associated genes. We generated the first loss-of-*Hsc70-5/mortalin*-function *Drosophila* model. The reduction of Mortalin expression recapitulates some of the defects observed in the existing *Drosophila* PD-models, which include reduced ATP levels, abnormal wing posture, shortened life span, and reduced spontaneous locomotor and climbing ability. Dopaminergic neurons seem to be more sensitive to the loss of *mortalin* than other neuronal sub-types and non-neuronal tissues. The loss of synaptic mitochondria is an early pathological change that might cause later degenerative events. It precedes both behavioral abnormalities and structural changes at the neuromuscular junction (NMJ) of *mortalin*-knockdown larvae that exhibit increased mitochondrial fragmentation. Autophagy is concomitantly up-regulated, suggesting that mitochondria are degraded via mitophagy. *Ex vivo* data from human fibroblasts identifies increased mitophagy as an early pathological change that precedes apoptosis. Given the specificity of the observed defects, we are confident that the loss-of-mortalin model presented in this study will be useful for further dissection of the complex network of pathways that underlie the development of mitochondrial parkinsonism.

## Introduction

Parkinson’s disease (PD) is a common movement disorder characterized by a progressive degeneration of dopaminergic (DA) neurons in the *substantia nigra* (SNc) [1]–[3]. Different genetic and environmental factors contribute to disease etiology. Mitochondrial dysfunction plays a profound role in the PD progression [4]–[6] and several genes associated with familial PD, such as *parkin*, *PTEN-induced putative kinase 1* (*PINK1*) and *DJ-1* have been proposed to regulate distinct features of mitochondrial function [5].

The ATPase domain-containing protein Mortalin is part of the molecular machinery that imports nuclear-encoded proteins into mitochondria, sorts them, and assists in their folding (for review, see [7], [8]). Mortalin function is regarded as critical for mitochondrial biogenesis; deletion of the yeast *mortalin* homolog *SSC1* is lethal [9], and knockdown of *mortalin* in immortalized human cells leads to growth arrest [10]. In contrast, overexpression of the mitochondrial chaperone Mortalin is sufficient to extend the life span of both *Caenorhabditis elegans* [11] and cultured human fibroblasts [12].

Mortalin acts as a buffer to prevent damage upon accumulation of dysfunctional, unfolded proteins in aging mitochondria. Unlike other heat shock proteins, Mortalin expression is not induced by heat shock; rather it is promoted by cellular stress, such as glucose deprivation, oxidative injury, radiation, and cytotoxins (for review, see [7], [8]).

Loss of Mortalin function is associated with PD. Decreased levels of Mortalin have been reported in advanced PD cases [13] and in the 6*-*hydroxydopamine rat PD model [14]. *Mortalin* variants were identified in Spanish [15] and German patients with PD [16]. *In vitro* studies revealed that PD-related Mortalin variants are associated with mitochondrial impairments, including morphological changes of mitochondria, increased reactive oxygen species production, and reduced mitochondrial membrane potential [16]. Importantly, these defects were exacerbated when the cells were challenged with proteolytic oxidative stress [16] and could be partially rescued by *parkin* overexpression [17].

*Drosophila melanogaster* has been successfully used to uncover molecular pathways underlying pathobiology caused by the loss of several PD-associated genes, including *pink1*, *parkin*, and *DJ-1* [18]–[22]. To generate a fly model for mortalin-associated PD, we used a targeted knockdown of *Hsc70-5,* the *Drosophila* homolog of human *mortalin*. We found that pan-neuronal silencing of *Drosophila mortalin* by RNA interference (RNAi) resulted in reduced fly viability, locomotion impairment, body posture defects, and reduced ATP levels. These phenotypes are highly reminiscent of defects described for other *Drosophila* models of PD-associated mitochondrial dysfunction [21], [23], [24]. In our *in vivo* model, loss of mitochondria precedes behavioral abnormalities and structural changes at the neuromuscular junction (NMJ) of *Drosophila* larvae. Mitochondrial fragmentation and degradation are very early defects that might be up-stream of later pathological events. This order of pathological events in the mortalin *Drosophila* model was then confirmed in a human *ex vivo* model. Our results suggest that mitophagy might be used as a biomarker for monitoring the predisposition to mitochondrial Parkinsonism.

## Materials and Methods

### *Drosophila* Strains and Culture Conditions

All flies were raised on standard corn meal/agar medium. Transgenic fly stocks were obtained from the Indiana University Stock Center (Bloomington, IN, USA), unless otherwise noted. Transgenic RNAi stocks were obtained from the VDRC stock center: *w^GD30033^, khc^GD44337^*, *mort^GD47745^*, and *mort^KK106236^* [25].

### Eye Phenotype Scoring

To examine the external *Drosophila* eye phenotype, adult flies were raised at 29°C under 12-h day/night cycles. For analysis, we used frozen flies that were not stored longer than 6 d at −20°C to avoid changes in the hue of the eye. Before taking images, flies were thawed and dried at room temperature for 10–15 min. Images were obtained with a DCM510 (ScopeTek, Hangzhou, P.R. China) camera mounted on a Zeiss Stemi 2000 stereomicroscope (Carl Zeiss, Oberkochen, Germany).

### Immunocytochemistry and Microscopy

The size-matched mid-L3 larvae were dissected and stained essentially as previously described [26]–[28]. Larvae carrying native GFP or mRFP constructs were fixed for 3 min (4% paraformaldehyde in phosphate-buffered saline) instead of 10 min. The goat α-horseradish peroxidase (HRP)-Cy3 antibody was obtained from Dianova (Hamburg, Germany).

The larval filets were imaged with a Zeiss LSM 710 Confocal Microscope using a 63×Plan-Apochromat 1.4 N.A. oil objective. The voxel dimensions (x/y/z) were 100×100×500 nm. The pinhole size was 1 Airy Disc. The images were processed essentially as previously described [29]. In brief, images were scaled by a factor of 2 before Gaussian blur filtering was applied (pixel radius = 2). Gamma values were not adjusted unless otherwise indicated. For quantitative comparisons of intensities, common settings were chosen to avoid oversaturation in any of the genotypes. Image processing was performed using Image J Software Version 1.43e (National Institutes of Health, Bethesda, MD, USA).

### Quantification of Mitochondria and Autophagosomes

We used the circularity, the inverse of the form factor, to assess the shape of mitochondria. The circularity of an object approaches 1 the more circular it is. It approaches 0 the more branched or complex the object is. Circularity was measured using ImageJ. Mitochondria with circularity >0.8 were defined as “round.” Images were thresholded to allow for semi-automated segmentation, counting, and classification of mitochondria using ImageJ Version 1.43e.

ATG8-mRFP, a widely used marker for autophagosomes [30], was used to quantify autophagosome abundance and size at the *Drosophila* NMJ. In the absence of autophagy, ATG8-mRFP is diffusely distributed in the cytoplasm. Autophagy induction leads to the recruitment of ATG8-mRFP into different sized puncta. While large ATG8-mRFP positive puncta generally represent autolysosomes, small puncta frequently did not overlap with lysotracker labeling, suggesting that they represent early autophagosomal structures [30]. We used a threshold to differentiate autophagosomes (Figure S1, arrowheads) from the diffuse cytoplasmic ATG-mRFP signal (Figure S1, arrows). The signal intensity observed in autophagosomes is generally 1.5–3-fold higher than the background. To improve visualization, we either used the false color look-up table “Green-Fire-Blue” (compare Figures S1A and B) or displayed autophagosomes after elimination of the cytosolic background by adjustment of brightness and contrast followed by an adjustment of Gamma values to 0.75 (compare Figures S1A and C).

### Behavioral Analysis

The walking behavior of 1-day-old female flies was assessed as previously described [31]. Flies were assayed three times in 15×15-cm petri plates. Each trial lasted 30 s. Individual trials were spaced at least 30 s apart. Walking ability was assessed by recording the number of crossings of 1×1-cm square grid lines marked on the bottom of the plate. At least 25 flies from each genotype were individually tested. Flies were raised and assayed at 18°C.

Climbing assays were conducted as previously described [32]. On the 6^th^ day after egg laying (AEL), larvae were transferred from 18°C to 25°C to induce expression of the UAS-constructs. Motor function of 4-day-old male flies was monitored by analyzing their ability to climb 6 cm within 14 s. A successful trial was scored as 1, and a non-successful trial was counted as 0. Each fly was assessed three times to calculate the average climbing score. At least 40 flies per genotype were analyzed.

The righting assay was preformed essentially as previously described [33]. Size-matched mid L3 larvae were collected from food and adapted to experimental conditions as previously described [28]. Next, larvae were placed upside down on the agar plate to measure the total time required to reposition the body posture to the ventral side down and for initiating the first contraction wave. At least 20 larvae of the same sex were analyzed per genotype. Each larva was assayed three times. The average righting time per larva was used for further analysis. N represents the number of larvae analyzed.

### Longevity Assay

Flies were maintained at 18°C in single-sex groups of no more than 15 flies per group. No anesthesia was used in the longevity experiments.

### Statistical Analysis

Statistical significance was assessed as previously described [32]. For the behavioral experiments and NMJ analyses, “n” represents the number of flies and number of NMJs assayed, respectively. *p-*values <0.05 were considered to be statistically significant. Data are expressed as means ± standard error values (*p<0.05, **p<0.01, ***p<0.001).

### ATP Measurements

ATP levels were measured in head homogenates using a luciferase-based bioluminescence assay. Five heads of female flies were homogenized in 6 M guanidine-HCl and frozen in liquid nitrogen. Next, samples were boiled for 3 min, cleared by centrifugation at 14,000 g for 5 min, and diluted to measure protein concentration (1∶10 diluted samples, Bradford Assay Kit, Sigma, St. Louis, MO, USA) and ATP level (1∶2,000 diluted samples, ATP Determination Kit Sensitive Assay, Biaffin GmbH & Co KG, Kassel, Germany). ATP levels were normalized to the protein concentration.

### Ethics Statement and Analysis of Human Cells

We obtained skin biopsies from two offspring of a PD patient, one carrying the heterozygous A476T *mortalin* variant and one representing the wild-type sibling control. The A476T variant carrier did not show any signs of PD at the time of biopsy. The participants provided written informed consent to participate in this study. The study was approved by the ethics committee of the Medical Faculty, Eberhard Karls University Tübingen, Germany. No minors/children participants were involved in the study.

Human fibroblasts were cultured, fixed, and analyzed essentially as previously described [16]. The passage number of fibroblasts was less than 10 for all experiments. Only fibroblasts with the same passage number were taken for experiments. For visualization of lysosomes and mitochondria, cells were incubated for 15 min in 100 nM Lysotracker Red DND-99 (Invitrogen, Carlsbad, CA, USA) or 100 nM MitoTracker Green FM (Invitrogen), respectively. Secondary antibodies were purchased from Molecular Probes (Invitrogen) or Zymed (San Francisco, CA, USA). Hoechst 33342 (Molecular Probes) was used to stain nuclei. Images were analyzed by Zeiss software AxioVision 4.6 and Image J Software Version 1.41o.

## Results

### Mortalin is Important for Neuronal Viability

**Figure 1:**
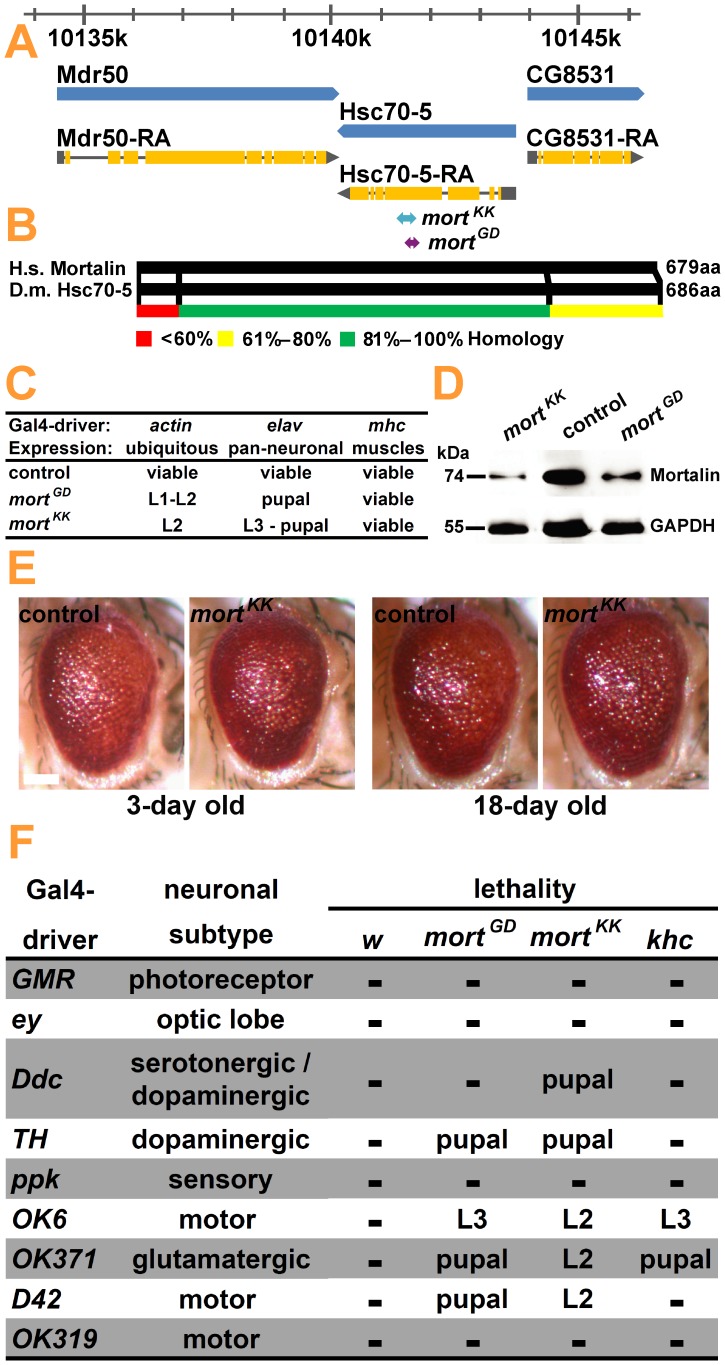
*Hsc70-5* (*CG8542, mortalin*) is a *Drosophila* homolog of the PD-associated gene *mortalin*. (**A**) The genomic organization of *Hsc70-5* (*CG8542, mortalin*) located on the second chromosome at cytological position 50E6. Genes and transcripts are displayed in blue and gray/yellow, respectively. Coding exons are depicted as yellow boxes, the 5′-UTR and 3′-UTR are shown as a gray box and a gray triangle, respectively. The exact sequence location (2R:10,140,103…10,143,697 [−]) is given at the top of the panel. *mortalin* expression was repressed using two UAS-RNAi stocks named *mort^GD47745^* (*mort^GD^*) and *mort^KK106236^* (*mort^KK^*). In *mort^GD^* (purple arrow) and *mort^KK^* (cyan arrow), 303-bp and 415-bp-long hairpin RNAs directed against gene fragments located to two partially overlapping domains in the fifth exon of *mortalin* were expressed. These double-stranded RNAs are processed into short siRNAs that are predicted to induce *mortalin* mRNA degradation. (**B**) *Drosophila* Mortalin (black box) has a high sequence similarity with human Mortalin. The 686-amino acid-long *Drosophila* Mortalin protein shares overall 73% identity and 84% similarity with the 679-amino acid-long human Mortalin. The percent homology, color coded in the bottom panel, between human and *Drosophila* mortalin is the highest in the central domain of the protein. (**C**) The ubiquitous and pan-neuronal knockdown of *mortalin* resulted in larval and pupal lethality, while *mortalin* knockdown in muscle did not impair viability. (**D**) The protein level of Mortalin in the ventral nerve cord (VNC) of mid third instar larvae was measured by western blot upon pan-neuronal expression (elav-GAL4, 29°C) of *mort^GD^* and *mort^KK^* (**E**) Eye-specific knockdown of *mortalin* did not cause visible defects in the external adult eye of the young and ageing flies. All the flies carrying the induced RNAi constructs were raised at 29°C. Scale bar: 0.1 mm (**F**) *Mortalin* deficiency in DA neurons is lethal, whereas GMR- and ey- driven expression of *mortalin^RNAi^* does not affect viability. Knockdown of *mortalin* in DA neurons using Ddc- or TH-GAL4 resulted in lethality during larval or pupal stages; no effect was seen following knockdown in sensory neurons. *mortalin* knockdown led to lethality with most GAL4 drivers that induce expression in motoneurons (OK6-, OK371-, D42-GAL4).

Mortalin is a highly conserved mitochondrial heat shock protein. We identified *Hsc70-5* (CG8542) as the fly ortholog of human *mortalin* (Figure 1A) that shares 73% identity and 84% similarity (Figure 1B). We utilized two transgenic RNAi stocks [25] targeting *mortalin*: *UAS-mortalin-RNAi^GD47745^ (mort^GD^)* and *UAS-mortalin-RNAi^KK106236^ (mort^KK^)* (Figure 1A, purple and cyan arrows) to examine the physiological consequences of its knockdown in *Drosophila*. The functionality of the RNAi constructs was assessed by ubiquitous (act-5C-GAL4) silencing of *mortalin* expression. Consistent with previous reports highlighting the importance of *mortalin* for mitochondrial function and cell viability [16], [17], [34], ubiquitous inactivation of *mortalin* expression was lethal at early larval stages (Figure 1C). Neurons are particularly vulnerable to mitochondrial impairments. Consistently, pan-neuronal (elav-GAL4) silencing of *mortalin* expression (elav*>mort*) was lethal in the late larval or early pupal stage, whereas RNAi-mediated *mortalin* silencing in muscle (Mhc-GAL4) did not affect viability. Analysis of Mortalin levels in the ventral nerve cords of the third instar larvae confirmed that both constructs efficiently suppressed *mortalin* expression, with a stronger reduction in protein level due to elav*>mort^KK^* activation (Figure 1D).

### Loss of *mortalin* in DA Neurons is Lethal

Motor symptoms in PD are primarily attributed to the progressive loss of DA neurons. Non-movement symptoms of PD, such as anxiety, depression, memory loss, and dementia, are thought to be caused by degeneration of noradrenergic, serotonergic, and cholinergic neurons [35]. To test the vulnerability of various neuronal sub-types to Mortalin loss, we employed different GAL4 drivers to express *mort^GD^* and *mort^KK^*. RNAi against the *white* gene (*white^RNAi^*) was used as a negative control, and RNAi against the hereditary spastic paraplegia (HSP)-related gene *kinesin heavy chain* (*khc^RNAi^*) was used as a specificity control. Gene expression was silenced in the following neuronal sub-types: (1) photoreceptor neurons; pigment cells, and neurons of the optic lobe, mushroom body, medulla cortex, lateral horn, and pars intercerebralis (GMR-GAL4, ey-GAL4); (2) DA and serotonergic neurons (Ddc-GAL4, TH-GAL4); (3) sensory neurons (ppk-GAL4); and (4) glutamatergic and motoneurons (OK6-GAL4, OK371-GAL4, D42-GAL4, OK319-GAL4). Photoreceptor neuron degeneration was scored on the basis of structural abnormalities on the external surface of the eye, such as the formation of black lesions and changes in eye pigmentation. Strong degeneration following the expression of toxic proteins using GMR-GAL4 might induce pupal lethality [36].

GMR*>mort^KK^* did not cause any morphological changes of the external eyes upon fly aging (Figure 1E). Neither ey- nor GMR-GAL4 induced *mortalin* silencing that resulted in lethality (Figure 1F). Knockdown of *mortalin* in DA neurons using Ddc- or TH-GAL4, but not in sensory neurons, resulted in lethality during the larval or pupal stages. These results suggest that Mortalin might be particularly important in DA neurons.

However, a direct comparison of phenotypes was complicated by variations in knockdown efficiency among different GAL4 drivers: for instance *mortalin* silencing in motoneurons driven by OK6-, OK371-, and D42-GAL4, but not by OK319-GAL4, resulted in lethality. A similar lethality pattern among different motoneuron drivers was observed upon inactivation of the HSP-related gene *khc*; however the silencing of *khc* expression in DA neurons did not affect viability (Figure 1F).

**Figure 2:**
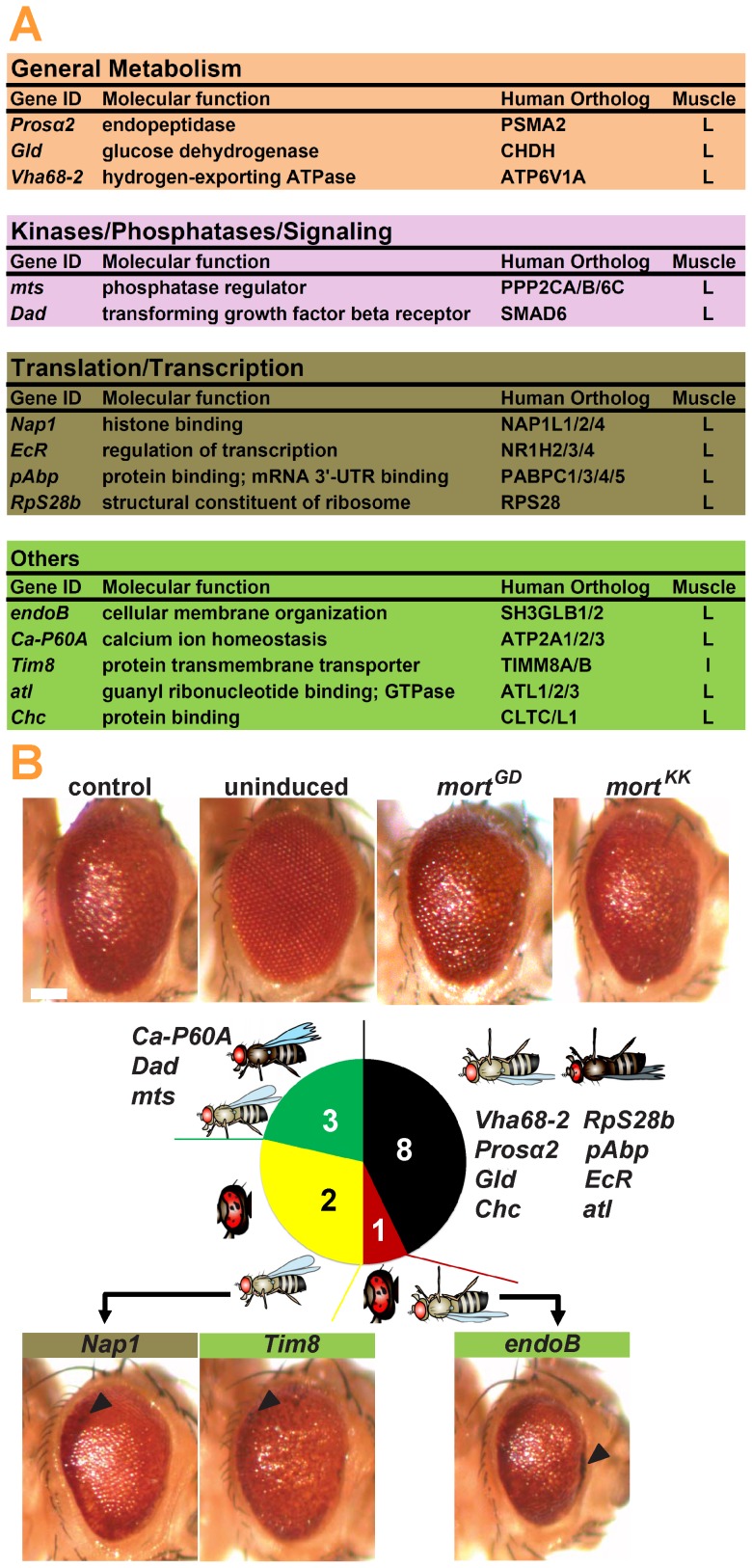
Analysis of the effects of housekeeping gene knockdown in *Drosophila* eye. (**A**) The eye-specific knockdown of *Drosophila* housekeeping genes resulted in diverse phenotypes. Examination of eyes revealed the effect of GMR-GAL4 driven RNAi silencing at 29°C. (**B**) GMR*>mort^KK^* did not cause degeneration in the external eyes of adult flies compared to the GMR-GAL4 (control), which displayed minor basal toxicity compared to uninduced flies (*mort^KK^*). The eye-specific inactivation of some *Drosophila* housekeeping genes induced strong degeneration. The arrowheads point to the black lesions indicative of necrosis. Scale bar: 0.1 mm.

To systematically assess whether DA neurons are particularly vulnerable to the loss of *mortalin,* 14 housekeeping genes were screened for defects following GMR- and TH-GAL4 induced knockdown (Figure 2A). All the RNAi-constructs had previously been validated for efficacy using mef2-GAL4 [37]. All the selected genes have human orthologs and cover a broad range of molecular functions, such as metabolism, cytoskeleton organization, signaling, translation, and transcription. Eight of the examined RNAi lines induced lethality upon expression under both TH- and GMR-GAL4 drivers. Only one of three RNAi constructs that induced eye degeneration caused lethality upon expression in DA neurons (Figure 2B). If the effects of *mortalin* knockdown were simply due to the higher efficacy of TH-GAL4 compared with GMR-GAL4, then one could find a gene whose silencing in the eyes and DA neurons would be reminiscent of the lethality pattern induced by *mortalin* inactivation. However, none of the three genes that failed to produce a clear phenotype in the eye upon GMR-induced silencing were able to cause lethality following knock down in DA neurons (Figure 2B). We thus concluded that the vulnerability of DA neurons to the loss of *mortalin* is unlikely to be an artifact.

Our next step was to investigate the cellular basis of the increased susceptibility of DA neurons to *mortalin* silencing. DA neurons might be particularly vulnerable to loss of mitochondrial function in general or susceptible to specific mitochondrial dysfunction caused by the loss of *mortalin*. To differentiate between these two possibilities, we selected a set of genes known to be important for mitochondrial function [38]. Using RNAi, the genes were inactivated in the eyes and TH-positive neurons [25]. We found that 3 of 10 RNAi constructs expressed under GMR-GAL4 caused pronounced degeneration in the eye, while none of the investigated RNAi constructs induced lethality upon TH-specific expression (Figure S2A,B).

We thus concluded that DA neurons are particularly susceptible to specific mitochondrial dysfunction caused by the loss of *mortalin*.

### Loss of *mortalin* Function Affects Body Posture and Locomotion

PD is a movement disorder characterized by muscular rigidity, tremor at rest, and postural instability [3]. *Drosophila* PD models exhibit locomotor and body posture dysfunctions that include abnormal wing posture, rigidity, and defects in flight and climbing abilities [19], [21], [23].

**Figure 3:**
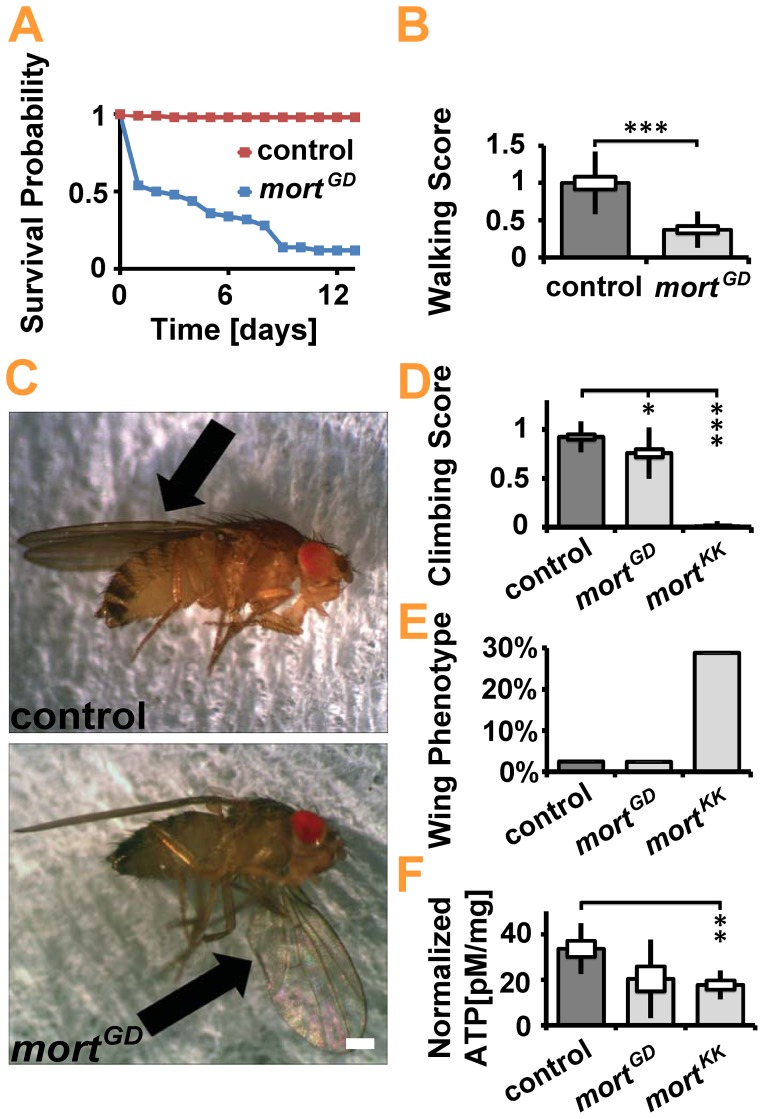
Pan-neuronal knockdown of *mortalin* caused behavioral defects and reduced adult *Drosophila* lifespan. (**A**) Kaplan-Meier survival curve recorded at 18°C. Lifespan reduction was detected upon pan-neuronal (elav-GAL4) *mortalin* knockdown. Female flies were examined. Statistical significance of the data was determined by a series of Mantel-Cox tests. (**B**) Walking tests showed that pan-neuronal *mortalin* silencing resulted in reduced locomotor function. All the flies were raised at 18°C. Statistical significance was determined using an unpaired, two-tailed Student’s t-test. (**C**) Characteristic wing posture phenotype caused by the weak pan-neuronal expression of *mortalin^RNAi^*. The top image displays the normal wings of control (elav*>white^RNAi^*) flies; the bottom picture shows the abnormal wing posture of elav*>mort^GD^*-expressing flies. All the flies were raised at 18°C. Scale bar: 0.25 mm (**D**) Climbing tests were used to assess locomotor behavior. Statistical significance was determined by using a Kruskal-Wallis H-test followed by a Dunn’s test for comparisons among multiple groups. (**E**) The characteristic wing posture phenotype caused by the pan-neuronal expression of *mortalin* RNAi. Pan-neuronal *mortalin* silencing in *mort^KK^* resulted in an increased wing phenotype percentage. (**F**) ATP level was measured in the heads of 4-day old female flies. Statistical significance was determined using a Kruskal-Wallis H-test followed by a Dunn’s test for comparisons between multiple groups.

To test whether chronic reduction of *mortalin* expression caused similar symptoms in adult flies, we pan-neuronally expressed *mort^GD^* and *mort^KK^* at 18°C, a temperature at which the UAS/GAL4 system is less active. Under these experimental conditions, control flies have a mean life span of more than 50 days, the median life span of flies expressing elav>*mort^GD^* was reduced to 2 days, and no flies expressing elav>*mort^KK^* emerged. Ten days after emergence, most elav*>mort^GD^* flies had died, while essentially all controls were still alive (elav*>mort^GD^*: 86% mortality, n = 50; control: 1% mortality, n = 100) (Figure 3A). Reduced longevity and locomotion defects are common features reported in *Drosophila* models of neurodegenerative diseases. Pan-neuronal knockdown of *mortalin* strongly affected the body posture and locomotion of elav*>mort^GD^* flies.

Climbing assays are commonly used to test locomotion, however the impaired body posture and overall weakness of the elav*>mort^GD^* flies required us to assess locomotor deficits using a less challenging assay that quantifies voluntary locomotion on a horizontal surface. Walking assays revealed a substantial impairment of motor capacities in 1-day-old *mortalin* knockdown flies (elav*>mort^GD^*: walking score 1±0.08; control: walking score: 0.25±0.05; n = 25 each; p<0.001) (Figure 3B). This locomotor disability worsened upon aging, suggesting rapid progressive degeneration (compare 5-day-old flies, Movies S1, S2). We found that 5-day-old flies generally neither moved nor climbed.

Body posture defects observed upon *mortalin* knockdown included abnormal wing posture: one or both wings were down-turned or held erect (Figure 3C). The abnormally down-turned posture of one wing (Figure 3C) is the most commonly observed phenotype in 1-day-old female flies. To bypass the early lethality of elav*>mort^KK^* we suppressed *mortalin* expression pan-neuronally starting 6 days AEL by using the GAL4/GAL80 system. Analysis of 4-day-old elav,tub-GAL80*>mort^KK^* flies confirmed the locomotion (Figure 3D) and body posture (Figure 3E) defects observed in elav*>mort^GD^* flies. Of note, the wing posture defects that developed upon *mortalin* knockdown were reminiscent of those in *pink1* mutant flies [39] and flies expressing *dOTC* that serve as a model for PD-related intra-mitochondrial protein misfolding [24]. We thus sought to investigate whether *mortalin* loss induces mitochondrial dysfunction by measuring total ATP levels in the heads of 4-day-old flies following conditional, pan-neuronal *mortalin* knockdown. Expression of elav,tub-GAL80*>mort^KK^* resulted in a 50% decrease in cellular ATP levels (Figure 3F). Given that mitochondria are the major source of ATP, our results suggest that loss of *mortalin* impairs mitochondrial ATP production.

### Loss of Mitochondria is an Early Pathological Manifestation in a Presymptomatic Loss of *mortalin* Function Model

Next, we sought to investigate the order of pathological changes during development. The differentiation between primary causes, secondary consequences, and compensatory adaptations becomes increasingly difficult as degenerative processes progress. The most common larval behavioral abnormalities caused by neurodegenerative processes include general larval locomotion impairment, sluggishness, or selective impairment of the posterior segments. For example, Parkin-deficient larvae are characterized by bradykinesia-like impairment in larval locomotion [40], and the expression of HSP-related mutations in *khc* leads to dystonic posterior paralysis (tail-flip phenotype) [32]. We thus analyzed crawling larvae with the aim of isolating *mortalin^RNAi^* larvae that do not yet display any impairment in locomotion or body posture.

**Figure 4:**
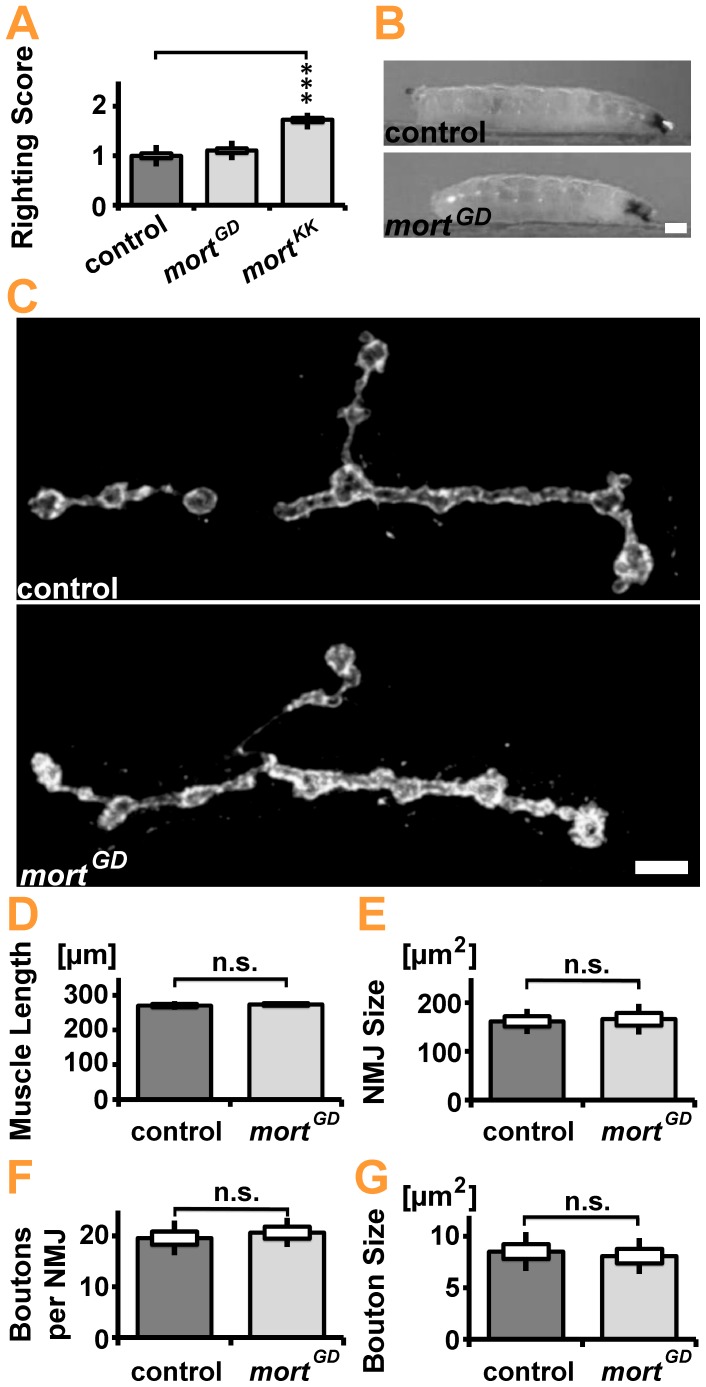
Quantification of synaptic terminals in *mortalin* knockdown larvae. (**A**) Larvae locomotor behavior and body posture control were assessed with the righting assay in 4-day old mid-L3 stage larvae. The average righting time is determined for larvae placed upside down on agar plate. Pan-neuronal *mortalin* silencing impaired locomotor function of *elav>mort^KK^* but not *elav>mort^GD^* larvae. Statistical significance was determined using a Kruskal-Wallis H-test followed by Dunn’s test for comparisons between multiple groups. (**B**) Analysis of larval crawling did not reveal any body-posture defect of 4-day old mid-L3 stage *elav>mort^GD^* larvae at rest or during locomotion. Scale bar: ∼0.25 mm (**C–G**) Confocal images of NMJ 4 at Segment A5 of the mid third instar larvae raised at 29°C. Visualization of neuronal membranes marked with HRP-Cy3 allowed assessment of NMJ morphology. Pan-neuronal expression of *mort^GD^* did not affect (**D**) muscle length, (**E**) NMJ size, or the number (**F**) or size (**G**) of synaptic boutons. Scale bar: 5 µm. Statistical significance was determined using an unpaired, two-tailed Student’s t-test.

Strong pan-neuronal expression of *mort^KK^* but not *mort^GD^* impaired larval locomotion and dynamic control of body-posture and position (Figure 4A) as determined by the righting assay [33]. elav*>mort^GD^* larvae displayed no abnormal body-posture at rest or during locomotion (Figure 4B, Movies S3, S4). Consistently, structural analyses of NMJs and synaptic boutons (Figure 4C) revealed no changes in NMJ size or bouton number or shape (Figure 4D–G). We thus concluded that elav*>mort^GD^* larvae might be considered as presymptomatic.

**Figure 5:**
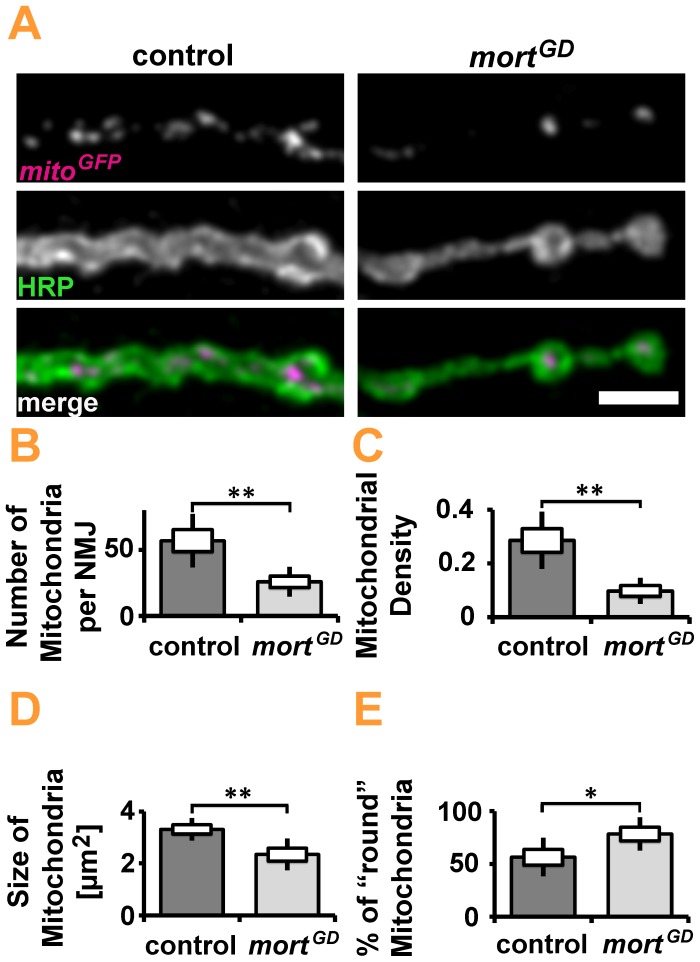
Quantification of mitochondria in *Drosophila* larvae upon silencing of *mortalin* expression. (**A**) Confocal images of synaptic boutons in control (elav*>white^RNAi^*) and elav*>mort^GD^* larvae. The membrane marker HRP-Cy3 is shown in green, and native fluorescence of *mito-GFP* is shown in magenta. Scale bar: 5 µm. *mortalin* silencing significantly reduced (**B**) the number of mitochondria per NMJ, (**C**) the area fraction of the NMJ positive for mitochondria. Furthermore, the average size (**D**) of mitochondria was reduced, while the fraction of circular mitochondria (**E**) was increased. Statistical significance was determined using an unpaired, two-tailed Student’s t-test.

**Figure 6:**
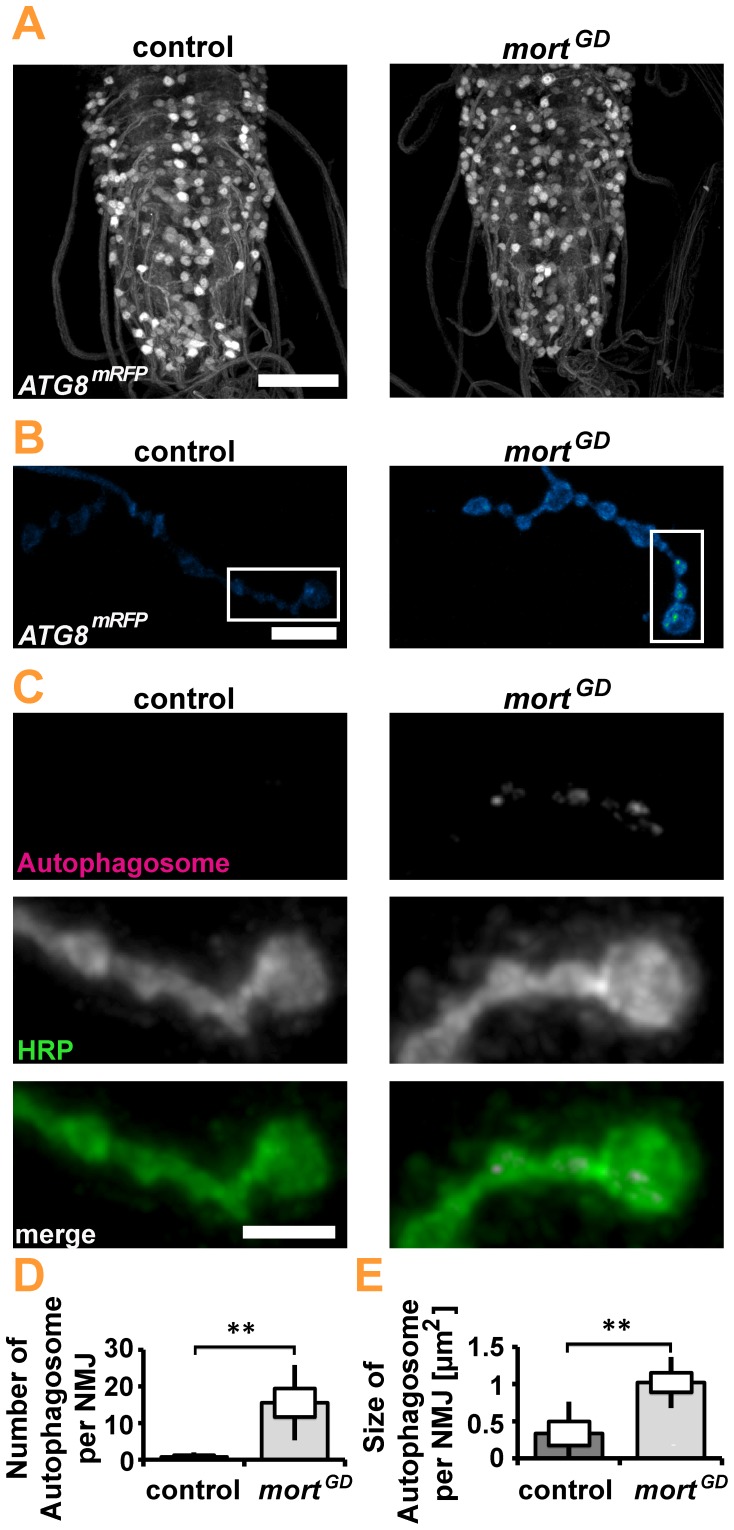
Pan-neuronal knockdown of *mortalin* induced autophagy at the larval NMJ. (**A**) *Drosophila* VNCs of control (elav*>white^RNAi^*) and elav*>mort^GD^* larvae labeled with the autophagosomal ATG8-mRFP marker. No obvious change in the ATG8-mRFP signal was detected upon *mortalin* knockdown. Gamma values were adjusted to 0.75 Scale bar: 50 µm. (**B**) Autophagosomes were detected as the strong accumulation of ATG8-mRFP signal at the *Drosophila* NMJ. The false color look-up table “Green-Fire-Blue” allows the separation of autophagosomes from the diffuse ATG8-mRFP signal. Scale bar: 10 µm. (**C**) Confocal images of synaptic boutons at NMJ 4 in control (elav*>white^RNAi^*) and elav*>mort^GD^* larvae. Neuronal membranes and autophagosomes are shown in green and magenta, respectively. Scale bar: 5 µm. (**D, E**) Statistical analysis revealed increases in ATG8-mRFP puncta abundance (**D**) and size (**E**). Statistical significance was determined by using an unpaired, two-tailed Student’s t-test.

**Figure 7:**
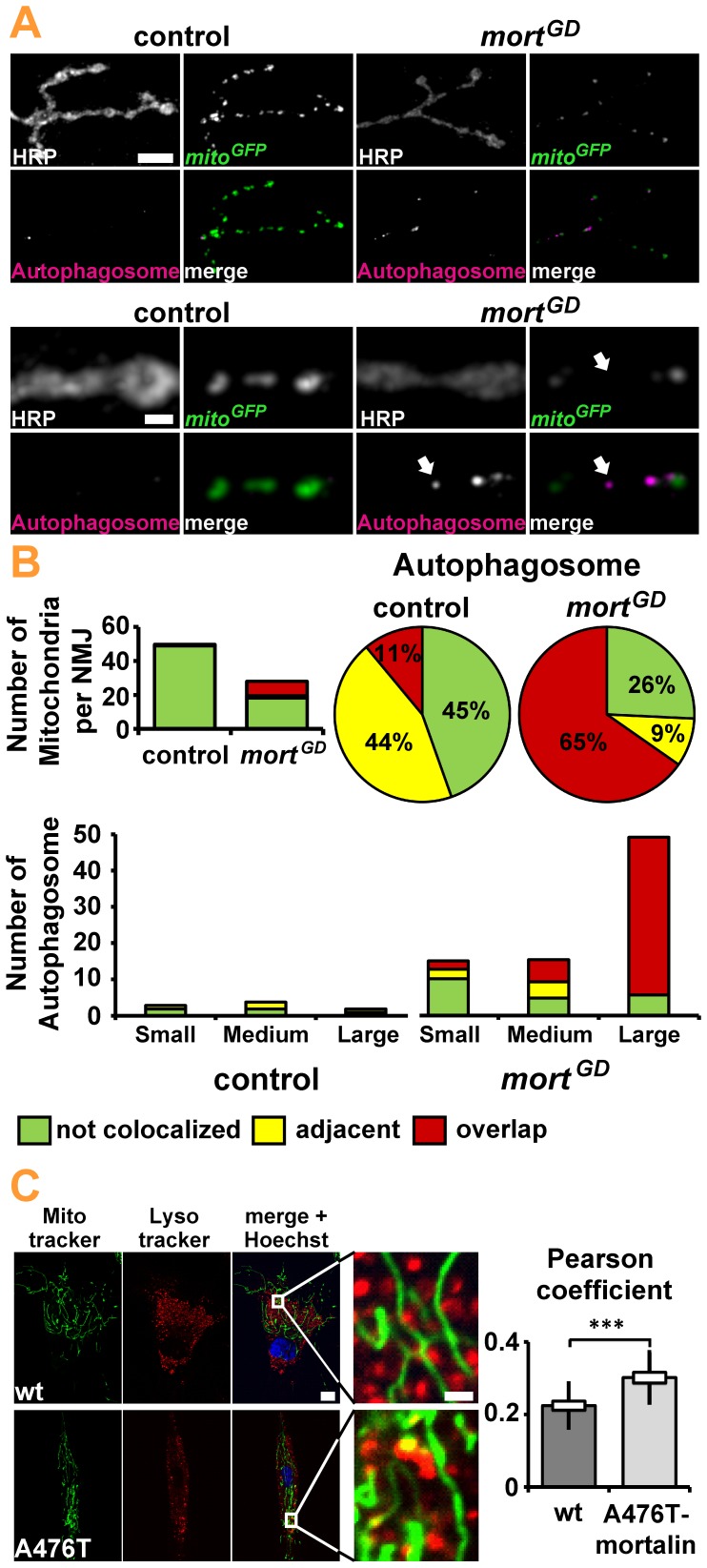
Loss of *mortalin* function induces mitophagy. (**A**) Confocal images of NMJ 4 at Segment A5 of the mid third instar larvae in control (elav>*white^RNAi^*) and elav>*mort^GD^* larvae. Neuronal membranes (HRP), autophagosomes, and mito-GFP are shown. In elav*>mort^GD^* larvae, mitochondria frequently co-localized with autophagosomes. Scale bar: 10 µm, Enlargement: 2 µm (**B**) The number of mitochondria and autophagosomes per NMJ is shown. Most autophagosomes in elav*>mort^GD^* larvae co-localized with mitochondria, either by being directly adjacent or overlapping. (**C**) In human fibroblasts (n = 56 cells) the mitochondrial-lysosomal colocalization was higher in cells from a carrier of the loss of *mortalin* function variant compared with cells from a healthy sibling control. Colocalization is indicated by a yellow signal due to overlapping Lysotracker red and Mitotracker green staining. Scale bar: 10 µm and 2 µm. Statistical analysis revealed a higher number of mitochondria colocalized with lysosomes in the mutant compared with control cells. Statistical significance was determined using an unpaired, two-tailed Student’s t-test.

Despite the absence of obvious changes in NMJ morphology, we detected significant reductions in mitochondria number, density, and size at the NMJs of elav*>mort^GD^* larvae (Figure 5A–D). We observed a significant increase in the percentage of round mitochondria (Figure 5E). Small round mitochondria are more easily engulfed by an autophagic membrane than large branched networks. We thus hypothesized that the loss of *mortalin* might induce mitophagy. To assess autophagy *in vivo,* we induced pan-neuronal (elav-GAL4) expression of the autophagy marker ATG8-mRFP, either in combination with *mortalin* or control RNAi [30]. We first investigated a potential generalized increase of autophagy in the ventral nerve cord of presymptomatic larvae. Pan-neuronal knockdown of *mortalin* did not cause any obvious increase in ATG8-mRFP levels in the central nervous system (CNS) of the affected larvae (Figure 6A). However, at NMJs (Figure 6B,C), the site at which marked changes in mitochondrial size, shape, and density were detected, we observed significant differences in autophagy. While almost no ATG8 puncta were present at control NMJs, many puncta were detected upon *mortalin* knockdown (Figure 6D). These puncta were larger than those present at control NMJs (Figure 6E). This increase in autophagy might suggest that mitochondria are degraded via mitophagy. Consequently, the autophagy marker ATG8-mRFP should co-localize with mitochondria. Consistently, 25% of all mitochondria were associated with autophagosomes in elav*>mort^GD^* larvae compared to less than 5% in controls (Figure 7A,B). A quantification of autophagosomes revealed that the organelles preferentially co-localize with mitochondria (Figure 7B), either by being directly adjacent (Figure 7A) or overlapping with mitochondria (Figure 7A). This preferential association with mitochondria was most pronounced for medium- and large-sized autophagosomes (Figure 7B), suggesting that these autophagosomes contribute the most to the phagocytosis of mitochondria. Small autophagosomes were generally not associated with mitochondria (Figure 7A, arrow; 7B). Thus, mitophagy induced by the loss of *mortalin* function might be one of the earliest cellular hallmarks of *mortalin* dysfunction-associated PD.

To test this hypothesis in human cells, we examined fibroblasts derived from a carrier of the A476T *mortalin* variant who did not show any signs of PD at the time of the biopsy. Previous reports using these fibroblasts revealed alterations in mitochondrial morphology compared to a healthy sibling control [16]. The impairments were reminiscent of the defects observed in the presymptomatic larvae, suggesting that these human cells are a good model for monitoring changes caused by the chronic reduction of Mortalin function at the early stages of PD. Consistently, no differences in basal level of apoptosis were detected between fibroblasts from the healthy sibling and the carrier of the A476T *mortalin* variant (unpublished results). In the latter cells, however, a strong decrease in mitochondrial mass was reported, suggesting that mitochondria are degraded via mitophagy (unpublished results) or suffer from decreased mitochondrial biogenesis. Next, we assessed mitophagy in the human *ex vivo* model to differentiate between these two possibilities. For this purpose, we calculated the Pearson coefficient to determine the correlation of fluorescence signals of lysosomal and mitochondrial structures. The fibroblasts from a carrier of the A476T *mortalin* variant revealed a higher degree of colocalization of mitochondria with lysosomes compared to the fibroblasts from a healthy sibling control (Figure 7C), indicating that dysfunctional mitochondria might be cleared via the autophagic pathway [41].

In summary, the analysis of our presymptomatic *in vivo* model and *ex vivo* data from human fibroblasts both suggest that loss of mitochondria may represent the earliest pathological change in the course of disease progression associated with impaired Mortalin function.

## Discussion

### Establishment of a *Drosophila* Model for PD-related Mortalin Dysfunction

Mortalin is essential for mitochondrial biogenesis, organellar quality control, and suppression of apoptosis (for review, see [7], [8], [34]). *Mortalin* variants that adversely affect mitochondrial function have been identified in PD patients, supporting the importance of Mortalin for mitochondrial function [16]. Pan-neuronally targeted *mortalin* knockdown led to a shortened lifespan, impaired walking and climbing, and abnormal wing posture. These phenotypes are reminiscent of symptoms observed in existing *Drosophila* PD models in which mitochondrial function is disrupted by either intra-mitochondrial proteolytic stress [24] or dysfunction of Pink1 or Parkin [21], [23].

Due to the similarity of the observed defects, we are confident that the model for loss-of-*mortalin*-related PD presented in this study will be useful for further dissection of the imbalance of complex molecular networks underlying the development of mitochondrial parkinsonism. It will be particularly interesting to use the powerful genetic tools available in *Drosophila* to validate known molecular interactions between *mortalin* and PD-associated genes, such as *PINK1*, *parkin*, and *DJ-1* (compare Refs [16], [42]–[44]), as well as to identify new Mortalin interaction partners.

### Vulnerability of DA Neurons to *mortalin* Loss

*mortalin* silencing in DA neurons generally affects whole organism viability more dramatically than *mortalin* knockdown in other cell types. Thus, DA neurons might be particularly vulnerable to the loss of mitochondrial function in general or susceptible to specific mitochondrial dysfunction caused by the loss of *mortalin*. Alternatively, a combination of specific and non-specific defects might contribute to the observed selective vulnerability. We favor the latter hypothesis for several reasons. Firstly, loss of *mortalin*, a broadly expressed essential gene, is expected to lead to a general impairment in cell viability. Both deletion of the yeast homolog *SSC1* and strong *mort^GD^* knockdown in *Drosophila* muscles using mef2-Gal4 are lethal [37], [45]. Consistently, *mortalin* knockdown leads to reductions in cellular ATP levels.

Although *mortalin* silencing in DA neurons caused early pupal lethality, the inactivation of other mitochondrial genes in the same neurons did not affect viability, suggesting that non-specific mitochondrial disturbances are not sufficient to adequately recapitulate the increased susceptibility of DA neurons observed upon *mortalin* silencing. Among the more than 150 distinct mitochondrial syndromes that together affect more than 1 in 5,000 live births, large heterogeneity of tissue- and organ-specific defects has been reported [46], [47]. Mitochondria are remarkably diverse to meet the specific demands of the specific cellular environment they face. For example, cardiac mitochondria are very robust, allowing them to maintain a constant ATP-to-ADP ratio over a fivefold workload range during exercise [48]. Thus, it is not surprising that mitochondria from two distinct organs are morphologically distinct and share “only” 75% common components [47], [49]. This heterogeneity explains selective pathology resulting from exposure to distinct mitochondrial toxins. While abuse of 1-methyl-4-phenyl-1,2,3,6-tetrahydropyridine (MPTP) causes selective DA degeneration and PD due to complex I inhibition [50], an epidemic blindness resembling Leber’s hereditary optic neuropathy was caused by combined folate deficiency and the consumption of home-made rum containing methanol that induced complex IV inhibition [51], [52]. It has been proposed that the lack of redundancy in mitochondrial quality control systems in DA neurons [53], [54] might render DA neurons more susceptible to mitochondrial dysfunction. *Mortalin* is of central importance for intra-mitochondrial quality control, suggesting that a combination of specific and non-specific disturbances might affect mitochondrial function in DA neurons, resulting in selective vulnerability of these cells.

But why are motoneurons also affected in our PD-model? Consistent with a previous report, the morphology and functional organization of SNc DA neurons might contribute to the increased vulnerability of these cells [55]. Although not primarily affected in PD, *Drosophila* motoneurons share the complex morphology of SNc DA neurons, necessitating complex trafficking and surveillance systems to supply healthy mitochondria to places of high-energy demand. *Drosophila* motoneurons are a simple, accessible, and well-characterized model system to decipher molecular mechanisms that underlie the susceptibility of neurons with extended morphology to impairments in mitochondrial trafficking and surveillance systems. Future research might additionally include a more detailed investigation of the less accessible *Drosophila* DA neurons.

### Reduction of Synaptic Mitochondrial Mass Precedes Apoptosis and Developing Motor Symptoms

Early pharmacological intervention is important for the success of any therapeutic approach. We were able to identify reduced mitochondrial mass as a pivotal cellular defect that precedes behavioral symptoms. This observation is consistent with previous studies on fibroblasts of one carrier of the PD-associated A476T *mortalin* variant [16]. As such, mitochondrial fragmentation might be caused by an imbalance between mitochondrial fission and fusion [6] or by increased mitophagy. Increased mitochondrial fission ought to increase the number of mitochondria. However, the opposite was observed: *mortalin* knockdown reduced the number of mitochondria at the NMJ. We thus propose that the accumulation of fragmented mitochondria is suppressed by increased autophagy [6]. Indeed, silencing of *mortalin* expression increased the abundance of autophagosomes and their co-localization with mitochondria, suggesting that mitophagy is an early cellular hallmark of Mortalin associated PD pathology.

Consistently, reduced mitochondrial mass (unpublished results) and increased co-localization of lysosomal and mitochondrial structures (Figure 7C) but no increased apoptosis (unpublished results) were observed in fibroblasts derived from a carrier of the PD-associated A476T *mortalin* variant.

Thus, the analysis of presymptomatic *Drosophila* larvae and the human *ex vivo* model both identify mitochondria loss as an early pathological change that precedes behavioral symptoms and apoptosis. Future research should address the perspective of monitoring mitophagy in patient-derived fibroblasts as a biomarker for predisposition to mitochondrial Parkinsonism.

## Supporting Information

Figure S1 **Quantification of autophagosomes in** ***Drosophila*** **mortalin knockdown larvae.** Confocal images of NMJs in control and elav*>mort^GD^* larvae. (**A**) The autophagosomes marker ATG8-mRFP (red) shows a diffuse staining in the entire NMJ (arrow). Autophagosomes (arrowheads) are detected by the strong accumulation of the ATG8-mRFP signal. Scale bar: 5 µm. (**B**) The false color look up table “Green-Fire-Blue” allows separating autophagosomes from background staining. (**C**) Alternatively, autophagosomes can be displayed by defining and removing the “non-punctate” through an appropriate indirect thresholding using the adjustment of image brightness and contrast. The same image adjustments are made for mutant and control NMJs.
(TIF)

Figure S2 **Effects of mitochondrial gene silencing in** ***Drosophila*** **eyes.** The knockdown of *Drosophila* mitochondria-related genes may cause degeneration in the external eyes upon the RNAi expression under GMR-GAL4 but failed to induce lethality while being driven by TH-GAL4 (*CoVIb, sesB, Trxr-1*). All the flies were raised at 29°C. The arrowheads point to the black lesions. Scale bar indicates 0.1 mm.
(TIF)

Movie S1 **Locomotion of** ***white^RNAi^*** **expressing flies.** The locomotion of a 5-day-old elav*>white^RNAi^* flies (18°C). The flies move normally.
(MPG)

Movie S2 **Locomotion of** ***mortalin^RNAi^*** **expressing flies.** The locomotion of a 5-day-old elav*>mort^GD^* flies (18°C). The flies are almost completely paralyzed, and they are unable to move or climb.
(MPG)

Movie S3 **Locomotion of** ***white^RNAi^*** **expressing larvae.** The locomotion of a 4-day-old L3 elav*>white^RNAi^* larva (29°C). The larva crawls normally.
(MPG)

Movie S4 **Locomotion of** ***mortalin^RNAi^*** **expressing larvae.** The locomotion of a 4-day-old L3 elav*>mort^GD^* larva (29°C). The larva crawls normally.
(MPG)
