# Supplementary figures and images for: Knockdown of Hsc70-5/mortalin Induces Loss of Synaptic Mitochondria in a Drosophila Parkinson’s Disease Model
Source: PLoS One. 2013 Dec 30;8(12):e83714. doi: 10.1371/journal.pone.0083714 (PMC3875477; doi:10.1371/journal.pone.0083714)

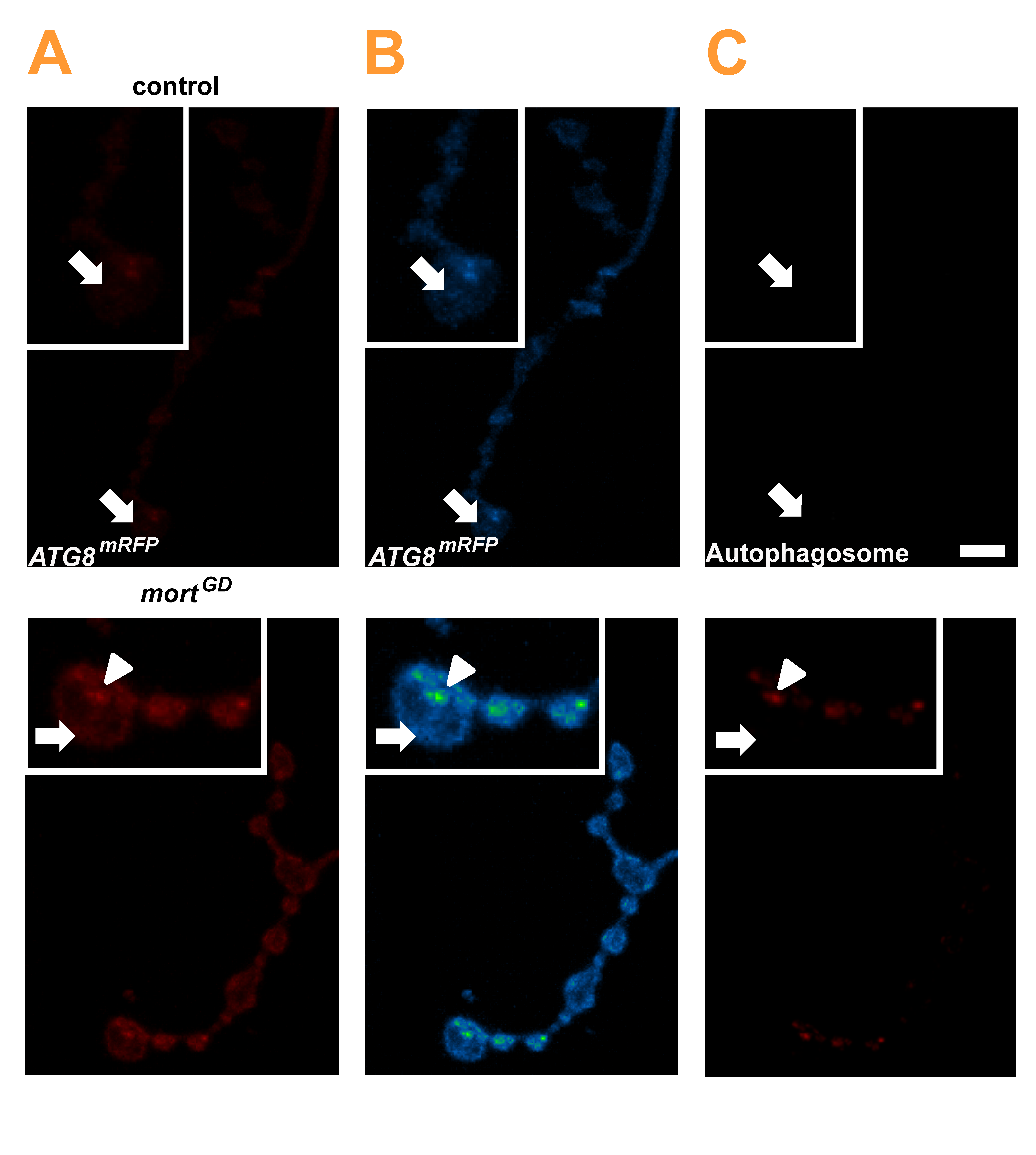

Supplement: Figure S1 — Quantification of autophagosomes in Drosophila mortalin knockdown larvae. Confocal images of NMJs in control and elav>mortGD larvae. (A) The autophagosomes marker ATG8-mRFP (red) shows a diffuse staining in the entire NMJ (arrow). Autophagosomes (arrowheads) are detected by the strong accumulation of the ATG8-mRFP signal. Scale bar: 5 µm. (B) The false color look up table “Green-Fire-Blue” allows separating autophagosomes from background staining. (C) Alternatively, autophagosomes can be displayed by defining and removing the “non-punctate” through an appropriate indirect thresholding using the adjustment of image brightness and contrast. The same image adjustments are made for mutant and control NMJs. (TIF) [file pone.0083714.s001.tif]

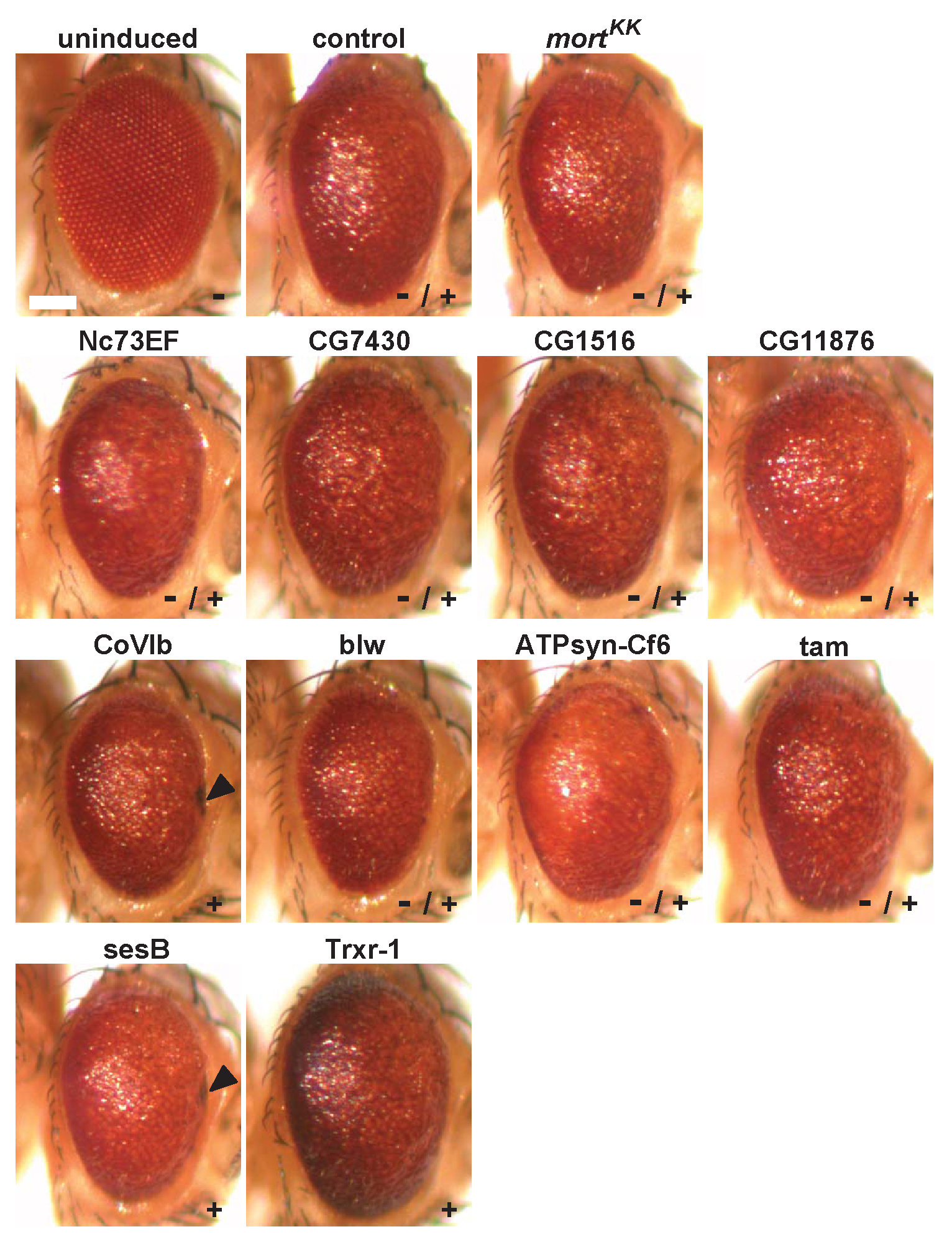

Supplement: Figure S2 — Effects of mitochondrial gene silencing in Drosophila eyes. The knockdown of Drosophila mitochondria-related genes may cause degeneration in the external eyes upon the RNAi expression under GMR-GAL4 but failed to induce lethality while being driven by TH-GAL4 (CoVIb, sesB, Trxr-1). All the flies were raised at 29°C. The arrowheads point to the black lesions. Scale bar indicates 0.1 mm. (TIF) [file pone.0083714.s002.tif]
